# Conceptualization and scale development for big data-based learning organization capability

**DOI:** 10.3389/fdata.2025.1596615

**Published:** 2025-06-19

**Authors:** Nesrin Alkan, Deniz Ersan Yilmaz, Bilal Baris Alkan

**Affiliations:** ^1^Faculty of Economics and Administrative Sciences, Akdeniz University, Antalya, Türkiye; ^2^Department of Educational Sciences, Akdeniz University, Antalya, Türkiye

**Keywords:** learning organizations, big data, organizational capability, digital transformation, scale development

## Abstract

**Introduction:**

In today's competitive business landscape, organizations must enhance learning and adaptability to gain a strategic edge. While big data significantly influences organizational learning, a comprehensive tool to measure this capability has been lacking in the literature. This study aims to develop a valid and reliable scale to assess big data-based learning organization capability.

**Methods:**

A two-phase research design was employed. In the first phase, Exploratory Factor Analysis (EFA) was conducted on data collected from 232 managers, identifying 22 items across three underlying factors. In the second phase, Confirmatory Factor Analysis (CFA) was applied to an independent sample (*n* = 128) to validate the scale's structure and its alignment with the theoretical model.

**Results:**

The EFA results revealed a clear three-factor structure, and the CFA confirmed the model's fit to the data, demonstrating good psychometric properties. The final BD-LOC scale shows high internal consistency and construct validity.

**Discussion:**

The BD-LOC scale provides organizations with a valuable tool to assess their big data-driven learning capabilities. It supports strategic decision-making, fosters innovation, and enhances operational efficiency. This study fills a significant gap in the literature and contributes to the effective implementation of digital transformation strategies in organizations.

## 1 Introduction

In today's business world, rapidly changing and increasing competitive conditions require companies and their employees to continuously improve their skills. In this environment, transforming into learning organizations has become a necessity for companies. Learning organizations are those that can access accurate information by leveraging the experiences of institutions and their employees, make strategic decisions by sharing this information effectively, and continuously renew these decisions. Such organizations should have five basic characteristics: systematic problem solving, testing innovative approaches, learning from experience, and rapid knowledge transfer (Garvin, 1993).

In recent years, big data has become an important tool in the business world, contributing to gaining a competitive advantage, predicting economic crises, strengthening decision-making processes, entering new markets, and ensuring operational efficiency. Learning from past successes is also a key component of learning organizations (Senge, 2006). Big data allows companies to perform predictive analyses and make more in-formed decisions. In this context, the combination of the concepts of learning organization and big data has strategic importance for companies.

Traditional learning processes focus on increasing the collective knowledge of organizations based on experiences and knowledge sharing among employees. This type of learning usually takes place through structured training, analysis of past experiences, and social interactions (Argyris and Schön, 1997). In contrast, big data-based learning refers to the data-driven, analytical, and real-time learning capabilities of businesses by extracting meaning from large, fast, and diverse data sources (McAfee and Brynjolfsson, 2012). Thanks to big data, companies can make innovative decisions and gain strategic flexibility by developing predictive models for the future, not limited to past experiences. In this context, big data-based learning offers a new paradigm that supports the development of learning organizations in the digital age by requiring technology integration, data analytics culture, and rapid knowledge transfer beyond traditional learning (Chen et al., 2012).

In recent years, the dynamic capabilities approach has become an increasingly important theoretical framework in the literature in evaluating the impact of digital transformation on organizational effectiveness. This approach emphasizes that businesses should continuously develop strategic competencies such as agility, innovation, and restructuring in digitalizing environmental conditions. Indeed, Volz et al. (2025) explained the impact of digital ecosystems on firm dynamics with this approach; they revealed that digital transformation is not only a technical application but also a strategic competency area. In the same vein, Al-Omoush et al. (2024) emphasized the cognitive and cultural dimensions of digital transformation by modeling the interaction between big data analytics and lean innovation through organizational learning. Similarly, León et al. (2024) presented strong empirical evidence on the strategic consequences of digital transformation by revealing the effects of big data analytics capabilities on innovative performance and overall business success. In light of these developments, it has become critical to understand how organizations can strategically transform through data-driven learning mechanisms. In this context, the concept of the learning organization, especially when empowered with big data capabilities, emerges as a fundamental structure for achieving sustainable digital transformation.

Various scale development studies in the literature aim to measure the ability to become a learning organization. For example, Armstrong and Foley (2003) aimed to identify the basic structures that facilitate the establishment and maintenance of a learning organization and provided a tool to systematically monitor progress toward a learning organization. Jashapara (2003) showed that double-loop learning and collaborative cultures have positive effects on organizational performance. Kim et al. (2017) emphasized that a learning organization positively impacts financial performance by increasing knowledge performance. Song et al. (2009a,b) demonstrated that the culture of a learning organization strengthens the relationship between interpersonal trust and organizational commitment. Xie (2020) stated that transformational leadership is a strong predictor for learning organizations. Törmänen et al. (2022) examined the concept of “system intelligence” in the context of learning organizations, presenting a new measurement tool to assess the ability of organizations to learn and develop in complex environments.

As a result of the literature review, many more articles were found on the learning organization scale. These studies are organized according to their creation dates and are presented in Table 1. However, a limited number of studies were found on the relationship between the concept of learning organization and big data (Miller, 2014). In the literature, studies on the role of big data in the transformation process of the learning organization are limited in number and are generally conducted with qualitative approaches. For example, BaşakBaşak (2024) contextually revealed the impact of big data on this transformation with case studies in the IT sector and emphasized the importance of sectoral dynamics. While such sector-focused studies provide important insights in the relevant fields, developing a scale that can be used validly and reliably for comprehensive evaluations of applications in different sectors makes a significant contribution to the literature in the field of measuring big data-based learning organization capacity. The Big Data-Based Learning Organization Capacity (BD-LOC) scale developed in this study responds to this need and provides a comprehensive and applicable tool to measure the contribution of big data to organizational learning.

**Table 1:** Scales developed for measuring learning organization capability.

**Title of the manuscript**	**Year**	**Authors**	**References**
Employees' perception of the learning organization	2001	Thomsen and Hoest	Thomsen and Hoest (2001)
Foundations for a learning organization: organization learning mechanisms	2003	Armstrong and Foley	Armstrong and Foley (2003)
Cognition, culture and competition: an empirical test of the learning organization	2003	Jashapara	Jashapara (2003)
The construct of the learning organization: dimensions, measurement, and validation	2004	Yang et al.	Yang et al. (2004)
A study on relationship among leadership, organizational culture, the operation of learning organization and employees' job satisfaction	2007	Chang and Lee	Chang and Lee (2007)
Measuring e-learning systems success in an organizational context: scale development and validation	2007	Wang et al.	Wang et al. (2007)
Learning organizations: diagnosis and measurement in a developing country context: the case of Lebanon	2008	Jamali and Sidani	Jamali and Sidani (2008)
The dimensions of learning organization questionnaire (DLOQ): a validation study in a Korean context	2009a	Song et al.	Song et al. (2009a)
The effect of learning organization culture on the relationship between interpersonal trust and organizational commitment	2009b	Song et al.	Song et al. (2009b)
An integrated scale for measuring an organizational learning system	2010	Jyothibabu et al.	Jyothibabu et al. (2010)
Dimensions of learning organizations questionnaire	2003	Marsick and Watkins	Marsick and Watkins (2003)
The effects of organizational structures and learning organization on job embeddedness and individual adaptive performance	2015	Kanten et al.	Kanten et al. (2015)
Learning organization and work engagement: the mediating role of employee resilience	2020	Malik and Garg	Malik and Garg (2020)
The impact of a learning organization on performance: focusing on knowledge performance and financial performance	2017	Kim et al.	Kim et al. (2017)
Measuring in-service teachers' attitudes towards inclusive education	2020	Clipa et al.	Clipa et al. (2020)
The impact of servant leadership and transformational leadership on	2020	Lei Xie	Xie (2020)
On the systems intelligence of a learning organization: introducing a new measure	2022	Törmänen et al.	Törmänen et al. (2022)

This scale will contribute to companies accelerating their digital transformation processes and developing more efficient strategies. Additionally, this study will discuss how the five basic characteristics of learning organizations, as suggested by Garvin (1993), can be measured in the context of big data, and how this tool can play a critical role in evaluating companies' progress toward becoming learning organizations. The research will also explore how big data can be used to become a learning organization and how the characteristics of learning organizations can be revealed more effectively. This research aims to contribute to both academic literature and business practices, helping companies manage their digital transformation processes more effectively and efficiently.

## 2 Materials and methods

### 2.1 Big data-based learning organization and related concepts

#### 2.1.1 Learning organization

A learning organization is defined as an organization's ability to develop itself and its members by continuously learning from internal dynamics or experiences in the external competitive environment, while systematically managing this process. This concept is crucial for organizations aiming to achieve sustainable success in the competitive, global business world (Schulz, 2002; Tsang, 1997; Örtenblad, 2001). In particular, the learning organization approach is seen as a strategic tool for enhancing both current and future company performance. Garvin (1993) listed the characteristics that learning organizations should have: systematic problem-solving, experimenting with new approaches, learning from their own experiences and history, learning from the experiences and best practices of others, and transferring knowledge quickly and effectively throughout the organization.

Being a learning organization provides various advantages to businesses. First, a more efficient operation is achieved by preventing the repetition of mistakes. The sustainability of strategic programs such as total quality management increases. The continuous learning process strengthens the collective intelligence and innovation capacity of the organization. This provides a competitive advantage by positioning organizations as leaders in their sectors. Additionally, the hidden potential of employees is revealed, transforming individual talents into corporate benefits. Employee loyalty is increased by creating a motivational work environment, which allows customer needs to be met more quickly and comprehensively (Wick and León, 1995).

#### 2.1.2 Big data

While data is generally defined as raw, unprocessed information, big data refers to extremely large, complex, and diverse datasets that exceed the processing capacity of traditional tools. These datasets typically contain information ranging from terabytes to petabytes, requiring specialized techniques for processing. Big data is typically defined by three basic components: volume (amount of data), variety (types of data), and velocity (speed of data flow). Additionally, some studies include other components such as accuracy, variability, and visualization as part of big data (Laney, 2001).

The concept of big data has gained rapid popularity in recent years and has significantly transformed the way businesses operate in an increasingly digital world. It is predicted that the volume of global data will double every 2 years (Mayer-Schönberger and Cukier, 2013). With increasing digitalization, more products and devices are connected to the internet, producing data. The Internet of Things (IoT) movement enables various products and devices to become data providers (McAfee and Brynjolfsson, 2012).

Big data involves not only large volumes of data but also the accurate collection, storage, processing, and transformation of this data into valuable information. Data from different sources, such as social media, sensors, and machines, play a crucial role in transforming business decision-making processes (McKinsey & Company, 2016). In this context, big data offers significant opportunities and strategic advantages for businesses. It accelerates decision-making, improves operational efficiency, and reduces costs. Data-driven analysis enables businesses to personalize customer experiences and predict market trends and risks more accurately. Moreover, big data enables companies to gain a competitive edge and presents opportunities to create new business ventures. These strategic advantages help businesses operate more agilely, proactively, and efficiently (Davenport, 2014; McKinsey & Company, 2016; Mayer-Schönberger and Cukier, 2013).

#### 2.1.3 Relationship between learning organization and big data

Big data analysis offers significant advantages for learning organizations, with the potential to enhance competitiveness and boost revenues. Rapid changes in the global business environment, increasing competition, and shorter product cycles require companies to adapt quickly (Garvin et al., 2008). The big data revolution has further strengthened this trend by accelerating changes in business processes (Gabel and Tokarski, 2014).

To fully benefit from big data, organizations need to have an information-centered structure. This structure allows for more data-driven decisions and enables employees to develop operational, tactical, and strategic plans based on real data. A big data culture requires employees to promote data collection and ask the right questions during every customer interaction. However, changing organizational culture is a challenging process and cannot be accomplished easily.

Cultural change is essential to fully benefit from big data, and the development and implementation of strategies depend on individuals within the organization, particularly leaders. As decision-makers begin to understand how big data will be integrated into the organization and the benefits it will provide, their chances of developing a successful strategy increase. At this point, experts such as data analysts, data architects, and data scientists play an important role. Organizations need to train these experts and enhance their IT departments with big data technologies to stay ahead of future developments.

Learning from past experiences is a key characteristic of learning organizations. This process should align with big data, as big data experts successfully implement solutions by associating technical issues with business goals (Gabel and Tokarski, 2014). The five basic characteristics that learning organizations should have, as identified by Garvin (1993), can be achieved using big data, as follows (Widyaningrum, 2016):

(i) *Systematic problem solving:* Big data provides scientific solutions based on data in decision-making processes.
(ii) *Experimenting with new approaches:* Big data enables new ideas to emerge that improve production processes and reduce costs.
(iii) *Learning from their own experiences and past:* Big data enables organizations to develop new strategies by analyzing their past successes and failures.
(iv) *Learning from others' experiences and best practices:* Big data enables organizations to improve by analyzing information from external sources.
(v) *Transferring information quickly and effectively throughout the organization:* Information obtained through big data is shared effectively within the organization, facilitating rapid decision-making.

Learning organizations should develop digital strategies to integrate big data. Digital capabilities, decision support systems, and product development strategies should be established, with the Human Resources department playing a key role in managing these processes (Minelli et al., 2013). Learning organizations continuously update their business processes and goals to adapt to rapidly changing conditions. Digital units are essential for the successful execution of these processes. As a result, big data is a powerful tool for learning organizations to achieve their goals. By making continuous improvements in data selection and analysis, learning organizations will increase profitability and maintain their competitive advantage.

### 2.2 Scale development

Based on the characteristics of learning organizations determined by Garvin (1993), a scale was developed to evaluate not only whether companies are learning organizations but also how they utilize big data in their transformation processes. The scale aims to assess the progress companies have made toward becoming learning organizations in the context of big data use and to measure their alignment with the characteristics identified by Garvin. In this way, the potential for becoming a learning organization and the effect of big data on organizational learning can be analyzed in detail.

In the development process of this scale, an initial pool of 80 items was created in line with the relevant literature and theoretical framework in order to ensure content validity. First, while defining the structure to be measured, topics such as the concept of the learning organization, its characteristics and capabilities, the definition of big data, the benefits of using big data in business life, and the impact of big data on becoming a learning organization were discussed in detail. The items were meticulously examined to eliminate possible ambiguities, repetitions, and other flaws. Items with an item-total correlation of < 0.30, ambiguous or repetitive content were eliminated, and the scale was reduced to 55 items at this stage. The data collection period for this study took place between 06/09/2023 and 06/02/2024. The Content Validity Ratio (CVR) method developed by Lawshe (1975) was used to assess content validity. This pool of 55 items was assessed by a group of five experts, including field experts and measurement and evaluation experts. As a result of independent evaluations, items with a CVR value < 0.99 (i.e., those evaluated as “not necessary” by at least one expert) were removed. At this stage, the grammatical correctness and comprehensibility of the items were also checked.

Following expert evaluations, some items were removed from the scale because they measured similar constructs, and a final set of 45 items was created. In addition, in line with expert opinions, edits were made to expressions that caused problems in terms of conceptual overlap, linguistic comprehensibility, and terminological consistency. At the end of this evaluation process, the scale was reduced to 45 items, and the CVR value of all remaining items was calculated as ≥ 0.99.

In order to examine the surface validity of the scale, a preliminary application was conducted on a smaller group representing a larger population. At this stage, a face-to-face pilot application was conducted with 20 participants, including business owners and managers in Antalya. Feedback was collected on the order of the items, response time, and comprehensibility. Pilot application, it was determined that the scale was completed in 8 min, the order of the items and the level of comprehensibility were sufficient, and it was not necessary to remove or revise any item in the scale. The scale consisting of 45 items was applied to 232 businesses of various sizes operating in different sectors in Antalya province and exploratory factor analysis was conducted. As a result of the analysis, items with low factor loadings and weakening the model fit were removed and the final version of the scale was determined as 22 items. A second independent sample consisting of 128 businesses was taken for the structural validity of the scale and confirmatory factor analysis (CFA) was conducted. Conducting exploratory factor analysis (EFA) and CFA on separate samples is a widely accepted practice in scale development studies because it helps verify that the factor structure is not sample specific and increases the generalizability of the model. This approach also reduces the risk of overfitting (Worthington and Whittaker, 2006).

## 3 Results

### 3.1 Results of exploratory factor analysis

In this study, factor analysis was conducted to determine the factor structure of the elements representing the big data-based learning organization capability. In the study, survey data were collected from 232 managers (44 micro-scale, 81 small-scale, 43 medium-scale, and 64 large-scale business managers). Data on the demographic characteristics of the participants are summarized in Table 2.

**Table 2:** Demographic characteristics of the participants.

**Feature**		**Number**	**Percentage**
Gender	Female	107	46,1
	Male	125	53,9
Education Status	Middle school	1	0,4
	High school	37	15,9
	University	148	63,8
	Master's degree	37	15,9
	Doctorate	9	3,9
Position	Business owner	94	40,5
	Manager	96	41,4
	Assistant manager	42	18,1
Year of work	0–5	25	10,8
	6–10	65	28,0
	11–15	40	17,2
	16–20	27	11,6
	More than 20	75	32,3

When Table 2 is examined, 46.1% of the participants were female, 53.9% were male, and the majority of the participants were university graduates. The distribution of roles such as business ownership, management, and assistant management reflects the diversity of the business structure. In terms of work experience, 32.3% of participants had 20 years or more of experience.

During the exploratory factor analysis (EFA), items with corrected item-total correlations below 0.30 were initially eliminated. The factor structure was then analyzed using the promax rotation method and principal axis factoring extraction (Briggs and Cheek, 1986; Clark and Watson, 1995; Hair et al., 2009). As a result of the iterative process, items with factor loadings below 0.50 and cross-loadings above 0.40 were systematically removed (Kline, 1994; Hair et al., 2009). Thus, 23 items were eliminated and a scale consisting of 22 items was developed.

The KMO value, calculated to determine the suitability of the sample size for factor analysis, was found to be 0.933. According to Field (2009), KMO values above 0.9 indicate that the data are very suitable for factor analysis. Additionally, the Bartlett's sphericity test was significant (Approximate Chi-Square: 2,020.471, *p* < 0.001). The significance of the Bartlett's test further supports the acceptability of the analysis values. As a result of the exploratory factor analysis, a three-factor scale consisting of 22 items with eigenvalues >1, explaining 70.759% of the total variance, was developed. According to the analysis results; the first factor has an eigenvalue of 10.889 and explains 49.495% of the total variance, the second factor has an eigenvalue of 2.968 and explains 13.490% of the variance, and the third factor has an eigenvalue of 1.710 and explains 7.774% of the variance. The reliability analysis of the 22-item scale showed a Cronbach's Alpha coefficient of 0.951. Scale factors and related Garvin criteria and their characteristics are presented in Table 3.

**Table 3:** Scale factors, Garvin criteria, and their characteristics.

**Factor names of the BD-LOC scale**	**Garvin's criteria for a learning organization**	**Number of items**	**Range of factor loadings**
Big data-based learning	•Systematic problem solving •Transfer information quickly and effectively throughout the organization	9	0.672–0.944
Learning based on new approaches	•Experiencing new approaches	5	0.711–0.842
Experience-based learning	•Learning from their own experiences and past •Learning from others' experiences and best practices	8	0.660–0.853

The three factors obtained are consistent with the basic characteristics for learning organizations suggested by Garvin (1993). The “Big Data Based Learning” factor coincides with the characteristics of being able to transfer information rapidly and systematic problem solving, while the “Learning Based on New Approaches” factor represents the ability to experiment with innovative approaches.

The “Experience Based Learning” factor reflects the characteristics of individuals learning from both their own experiences and the experiences of others. These three factors reflect important components of organizational learning processes and are strongly related to existing theories in the literature. Table 4 shows the items of which each factor consists.

**Table 4:** Factor loading for the BD-LOC scale (22 items).

**Items**	**Factor 1**	**Factor 2**	**Factor 3**
There are experienced personnel who can analyze the collected data.	0.944		
Our business has appropriate technology for storing and analyzing data.	0.934		
We use advanced technology and analysis tools to further improve the use of big data in our business and strengthen the features of a learning organization.	0.823		
Business processes are improved by constantly monitoring the analysis and results of big data.	0.791		
Digital transformation activities are included in our business.	0.775		
Regular meetings are held to share and discuss big data analysis results in all departments of our business.	0.765		
Adequate precautions are taken regarding data security and privacy and our employees are made aware of this issue.	0.752		
Our employees are encouraged to make data-driven decisions.	0.714		
Big data analysis results are shared between different departments within the business, ensuring collaboration and learning.	0.672		
Customer feedback is used to quickly update and improve our products and services.		0.842	
New products and marketing strategies are tried.		0.829	
Efforts are made to improve existing products and services on a regular basis.		0.810	
New ideas that have not been tried in the business are allowed.		0.773	
Efforts are made to increase customer satisfaction and reduce complaints.		0.711	
The employee returning from training is asked to produce a project.			0.853
Within the scope of the personnel rotation program, personnel are relocated between units or branches of the enterprise at certain times.			0.818
Despite the workload, importance is given to education by allocating time.			0.814
In the business, personnel who receive and implement training are rewarded.			0.801
Employees are encouraged to receive training on data analytics topics.			0.792
In-service training is provided periodically.			0.745
A personnel who has achieved success in his own unit is assigned to improve a lagging unit.			0.706
In our company, our employees are provided with personalized training according to their needs.			0.660

Factor 1, Big data based learning; Factor 2, Learning based on new approaches; Factor 3, Experience based learning.

### 3.2 Results of confirmatory factor analysis

In the scale development process, confirmatory factor analysis (CFA) was applied to test the validity of the factor structure determined by explanatory factor analysis (EFA). In order to verify the structure of the scale on another sample, CFA was conducted on a group independent of the sample used in EFA. For this purpose, data was collected from 128 managers from an independent sample, completely on a voluntary basis. Demographic information of the participants is presented in Table 5.

**Table 5:** Frequencies and percentages of demographic characteristics of participants (Sample 2).

**Feature**		**Number**	**Percentage**
Gender	Female	72	56,3
	Male	56	43,8
Education status	High school	27	21,1
	University	74	57,8
	Master's degree	27	21,1
Position	Business owner	81	63,3
	Manager	19	14,8
	Assistant manager	28	21,9
Year of work	0–5	23	18,0
	6–10	38	29,7
	11–15	27	21,1
	16–20	14	10,9
	More than 20	26	20,3

According to the second application results, 56.3% of the participants are female and 43.8% are male. When the education levels are examined, it is seen that the majority of the participants (57.8%) are university graduates. In terms of their working positions, 63.3% of the participants are business owners, 14.8% are managers and 21.9% are assistant managers. When the working period is taken into consideration, it is remarkable that 21.1% of the participants have 11–15 years of experience. In addition, this sample includes data from 39 micro, 32 small, 18 medium, and 39 large-scale enterprises.

The three-factor structure was tested with confirmatory factor analysis (CFA). CFA was performed on the confirmatory sample consisting of 128 participants using the LISREL 8.80 program (Joreskog and Sorbom, 2006). The maximum likelihood method was preferred as the parameter estimation method. CFA aims to evaluate the degree to which the data obtained through the scale's goodness of fit indices are compatible with the model. The goodness of fit indices suggested by Kline (2016) were used in the evaluation of the measurement model. The confirmatory factor analysis (CFA) results support the structural validity of the scale and the three-dimensional factor structure. The goodness of fit measures of the CFA model were found to be ${\chi^{2}}_{\left(206\right)}$ = 412.06, *p* < 0.001, CFI = 0.97, TLI = 0.93, RMSEA = 0.08, SRMR = 0.070, NFI = 0.94. These values prove that the model provides a good fit with the data and the validity of the proposed structure. Table 6 shows the obtained goodness of fit indices and their acceptable limits (Schermelleh-Engel et al., 2003).

**Table 6:** Goodness of fit index.

**Goodness of fit index**	**CFA measurements**	**Acceptable limits**
χ^2^/df	2	< 3
CFI	0.97	≥ 0.95
TLI	0.93	≥ 0.90
RMSEA	0.08	≤ 0.08
SRMR	0.070	≤ 0.08
NFI	0.94	≥ 0.90

The findings support the reliability and validity of the big data-based learning organization ability scale. In order to evaluate the reliability and validity of the scale, composite reliability (CR) and average variance extracted (AVE) values were calculated. Composite reliability (CR) is a measure that evaluates the internal consistency of a factor and is generally used to determine the reliability of a factor. A value above 0.70 for the CR value generally indicates high reliability. Average variance extracted (AVE), on the other hand, measures the explanatory power of each factor. When the AVE value is generally 0.50 and above, the factor is considered to have a high level of convergent validity. In other words, a factor with an AVE value above 0.50 indicates that the relevant measurement tool represents the factor well (Fornell and Larcker, 1981). As a result of this analysis, CR and AVE values are given in Table 7.

**Table 7:** Reliability and convergent validity measures.

**Factor**	**CR**	**AVE**
Big data-based learning	0.93	0.60
Learning based on new approaches	0.90	0.65
Experience-based learning	0.93	0.61

**Figure 1:**
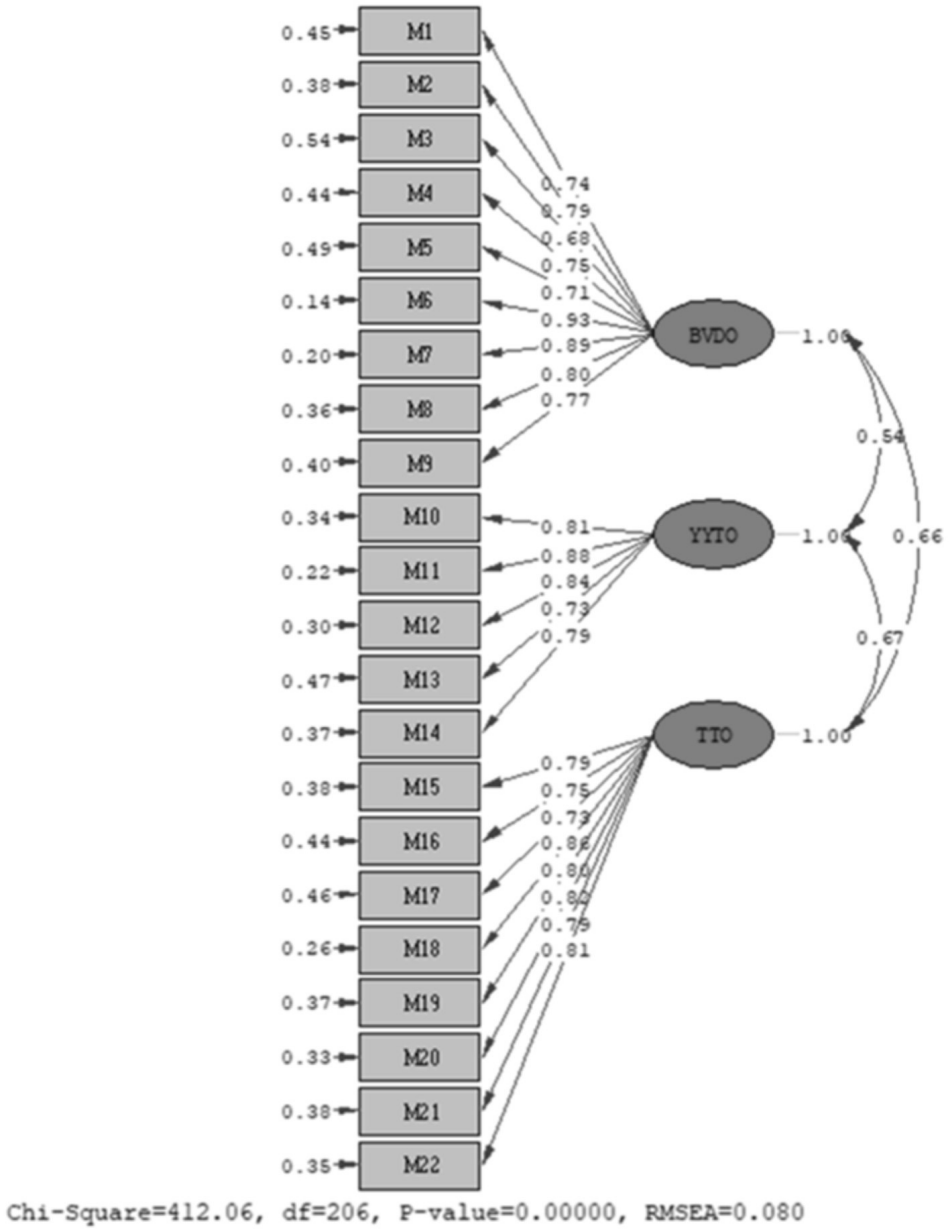
The results for the CFA model.

These results show that CR > 0.70 and AVE > 0.50 conditions are met for all three factors and therefore the scale has high reliability and validity. As seen in Figure 1, correlations between factors vary between 0.54 and 0.67. Statistically significant correlations were found between Factor 1 and other factors (Factor 2 and Factor 3) and between Factor 2 and Factor 3. These findings show that there are significant relationships between factors and that the scale has a valid structure. All these results reveal that both the structural validity and reliability of the 22-item three-dimensional scale are strong. The scale presents a systematic model representing the big data-based learning organization capability.

## 4 Conclusion

In this study, the big data-based learning organization capability (BD-LOC) scale was developed. The scale aims to measure the big data-based learning organization capacity of companies across three dimensions: big data-based learning, learning through new approaches, and experience-based learning. As a result of the analyses conducted with two independent samples, it was observed that the scale demonstrated high performance in terms of internal consistency reliability, construct validity, and convergent validity. These findings indicate that the BD-LOC scale is a reliable tool for evaluating the big data-based learning processes of companies.

The study makes a significant contribution to the limited body of literature combining the concepts of big data and learning organizations. The BD-LOC scale provides a useful tool for both academic research and business applications. Using this scale, managers can assess perceptions related to digital transformation processes and make more informed decisions regarding big data-based strategies. Additionally, this scale offers new opportunities to monitor the progress of companies toward becoming learning organizations and to develop big data-supported training programs.

The fact that our study was conducted with data obtained from managers working in businesses in different sectors in Antalya may limit the generalizability of the findings across cultures. In this context, future studies that apply the BD-LOC scale to different cultures and evaluate the measurement equality across cultures will contribute to the universal validity of the tool. Testing the BD-LOC scale in different sectors and organizational contexts provides an important basis for understanding the impact of sector-specific dynamics on big data-based learning practices. In addition, the Likert-type self-report scale used in the study is suitable for assessing the perceptions of the participants. In future studies, comparative analysis of the scale results with objective organizational data will also provide important contributions in terms of behavioral validity. On the other hand, the data obtained from this scale can be used to develop predictive models to determine whether businesses are big data-based learning organizations. Such models have the potential to improve both academic and practical use of the scale.

## Data Availability

The raw data supporting the conclusions of this article will be made available by the authors, without undue reservation.
